# Effects of chronic home radon exposure on cognitive, behavioral, and mental health in developing children and adolescents

**DOI:** 10.3389/fpsyg.2024.1330469

**Published:** 2024-02-26

**Authors:** Brittany K. Taylor, Haley Pulliam, OgheneTejiri V. Smith, Danielle L. Rice, Hallie J. Johnson, Anna T. Coutant, Ryan Glesinger, Tony W. Wilson

**Affiliations:** ^1^Institute for Human Neuroscience Boys Town National Research Hospital, Omaha, NE, United States; ^2^Center for Pediatric Brain Health, Boys Town National Research Hospital, Omaha, NE, United States; ^3^Department of Pharmacology and Neuroscience, Creighton University, Omaha, NE, United States

**Keywords:** radon, development, self-regulation, emotion-regulation, chronic exposure

## Abstract

**Introduction:**

It is well-established that chronic exposure to environmental toxins can have adverse effects on neuropsychological health, particularly in developing youths. However, home radon, a ubiquitous radiotoxin, has been seldom studied in this context. In the present study, we investigated the degree to which chronic everyday home radon exposure was associated with alterations in transdiagnostic mental health outcomes.

**Methods:**

A total of 59 children and adolescents ages 6- to 14-years-old (*M* = 10.47 years, *SD* = 2.58; 28 males) completed the study. Parents completed questionnaires detailing aspects of attention and executive function. We used a principal components analysis to derive three domains of neuropsychological functioning: 1) task-based executive function skills, 2) self-and emotion-regulation abilities, and 3) inhibitory control. Additionally, parents completed a home radon test kit and provided information on how long their child had lived in the tested home. We computed a radon exposure index per person based on the duration of time that the child had lived in the home and their measured home radon concentration. Youths were divided into terciles based on their radon exposure index score. Using a MANCOVA design, we determined whether there were differences in neuropsychological domain scores across the three groups, controlling for age, sex, and socioeconomic status.

**Results:**

There was a significant multivariate effect of radon group on neuropsychological dysfunction (λ = 0.77, *F* = 2.32, *p* = 0.038, η_p_^2^ = 0.12). Examination of univariate effects revealed specific increases in self-and emotion-regulation dysfunction among the youths with the greatest degree of chronic home radon exposure (*F* = 7.21, *p* = 0.002, *η*_p_^2^ = 0.21). There were no significant differences by group in the other tested domains.

**Discussion:**

The data suggest potential specificity in the neurotoxic effects of everyday home radon exposure in developing youths, with significant aberrations in self-and emotion-regulation faculties. These findings support the need for better public awareness and public health policy surrounding home radon safety and mitigation strategies.

## 1 Introduction

Exposure to environmental toxins is known to impact multiple aspects of cognitive, behavioral, and mental health (Trimble and Krishnamoorthy, 2000; Bornschein et al., 2006; Liu and Lewis, 2014; Rahman et al., 2021). Importantly, children are often more vulnerable than adults to the effects of toxic exposures and show some of the greatest immediate-and long-term neurocognitive consequences following exposure (Bearer, 1995, 2000; Falck et al., 2015). This is in part due to unique differences in the nature of toxic exposures among children relative to adults, with children generally having greater consumption-and absorption rates of numerous ingested and inhaled toxins due to their physical structure (e.g., proximity to floor), behavior (e.g., putting objects in the mouth) and physiology (e.g., gut absorption, breathing rate; Bearer, 1995; Grigg, 2004). Further, there is mounting evidence that the developing nervous system has a heightened vulnerability to toxic exposures given that the brain is highly plastic throughout childhood, and is thus readily influenced, for better or worse, by environmental inputs (Kalia, 2008).

There is a long history of research characterizing the neurotoxic effects of environmental exposures. For instance, lead exposure, which commonly occurs through ingestion, has been repeatedly linked to broad neurocognitive deficits spanning executive functions, emotional control, and sensorimotor processes (Silver et al., 2016; Ramírez Ortega et al., 2021). Other studies focusing more on inhaled toxins have also shown an array of effects. Common contaminants like household dust, which can contain plastics and flame retardants, have been linked with lower overall and verbal intelligence, attention deficits, poorer fine motor control, and greater externalizing symptoms in youths (Shaw et al., 2014; Zhao et al., 2022). Similar patterns of neuropsychological harm have been noted following other environmental exposures like chemicals common in household and agricultural care (Stein et al., 2013; Rauh and Margolis, 2016), as well as multiple forms of ambient particulate matter from common sources like vehicle exhaust (Guxens and Sunyer, 2012; Myhre et al., 2018). Surprisingly, despite the extensive literature exploring the impact of pollutants on neuropsychological health, some ubiquitous inhaled toxins have been seldom studied for their effects on developing neurocognitive abilities. One such toxin is radon.

Radon is a known carcinogenic substance, and serves as the leading cause of lung cancer among non-smokers globally (Darby et al., 2005; Vogeltanz-Holm and Schwartz, 2018). A radioactive byproduct of uranium decay in soil, radon gas can seep into buildings through cracks in the foundation caused by settling or deliberate openings like those for plumbing, and can build up to potentially dangerous levels (United States Environmental Protection Agency, 2003). In fact, 1 in every 15 homes across the United States is expected to test above the action limit of 4.0 pCi/L set by the U.S. Environmental Protection Agency; this is the carcinogenic equivalent of smoking 10 cigarettes per day (United Stated Environmental Protection Agency, 2016). This is of great importance considering that the home is the greatest source of indoor radon exposure for the majority of people (Stanley et al., 2019). Even so, many homes across the country remain untested and/or unmitigated, leaving a large portion of the population persistently exposed to this environmental toxin (Wang et al., 2000; Vogeltanz-Holm and Schwartz, 2018; Novilla et al., 2021). This is particularly alarming in light of recent work showing that indoor radon concentrations are actually increasing over time, and staying consistently high across seasons as home construction and the global climate continue to change (Stanley et al., 2019).

The effects of radon exposure are most commonly noted later in life. As stated previously, radon has been repeatedly linked to the incidence of lung cancer in adulthood (Chen, 2013). Beyond cancer, and perhaps more pertinent to the question of cognitive and mental health, recent epidemiological works suggest that radon exposure is associated with the incidence of neurodegenerative diseases, including Alzheimer’s disease and multiple sclerosis in older adults (Kempf et al., 2013; Zhang et al., 2022). Mechanistically, these impacts on the brain are believed to stem from a chronic, persistent process of environmental toxicity occurring over a lifetime impacting immune functioning, cellular health, and biochemical processes (Zhang et al., 2022). Given the long-term, cumulative nature of effects, there is increasing interest in understanding the early roots of such neuropsychological and physiological dysfunction seen in aging populations. In fact, a recent study demonstrated that youths with greater home radon exposure also tended to have higher levels of circulating biomarkers of inflammation (Taylor et al., 2023), which may be a precursor to systemic downstream effects. Despite such data, the field’s understanding of how radon exposure affects youths’ neuropsychological well-being remains extremely limited.

The purpose of the present study was to better characterize the potential impacts of everyday home radon exposure on cognitive, behavioral, and mental health in a sample of developing children and adolescents. We specifically assessed individual home radon exposure, including the concentration and chronicity of radon exposure in each participant’s home, alongside well-validated measures of cognitive functioning. Given the consistent links in the literature between psychological outcomes and exposures to other common environmental toxins, we hypothesized that increasing radon exposure would be broadly associated with greater levels of parent-reported problems in children’s mental functioning.

## 2 Methods

### 2.1 Participants

Children and adolescents who were part of an ongoing observational study of neurocognitive development were invited to participate in the current protocol. A total of 68 youths ages 6 to 14 years-old (*M* = 10.27 years, *SD* = 2.59; 33 male) consented to the study, and their families completed a home radon test kit. Exclusion criteria included history of head trauma, neurological disorder or other medical illness affecting brain function, current substance abuse, and standard neuroimaging exclusions (e.g., dental braces, other non-removable ferromagnetic materials on the body). Potential participants were not excluded on the basis of existing home radon mitigation systems. All parents of child participants provided signed informed consent, and youth participants gave signed assent to participate in the study. All procedures were approved by an Institutional Review Board and were compliant with relevant federal and international regulations.

### 2.2 Questionnaires

Parents were asked to complete a brief questionnaire when they began their home radon testing for the study. The custom-designed questionnaire asked for details about the construction of the home (e.g., how many stories, type of foundation, location of children’s bedrooms), how long the family had lived in the home, whether the home had ever been tested/mitigated for radon, and whether anyone in the home smoked cigarettes. The questionnaire was completed remotely at the parent’s convenience via the Collaborative Informatics and Neuroimaging Suite.^1^

In addition to the custom radon questionnaire, parents were asked to complete a battery of questionnaires detailing child- and household demographic information, as well as the parent’s perspective on their child’s cognitive, behavioral, and mental health. Parents completed the Barratt Simplified Measure of Social Status (Barratt, 2006), which assesses parental education and occupation to provide a numeric index of socioeconomic status (SES). Scores can range from 17 to 66, with higher scores indicating higher SES.

With respect to cognitive, behavioral, and mental health of the child, parents were asked to complete two neuropsychological questionnaires. First, the Behavior Rating Inventory of Executive Functioning, Second Edition (BRIEF-2; Gioia et al., 2000, 2015) is comprised of 86 items pertaining to a child’s executive control abilities. Parents rate on a 3-point scale (1 = “never,” 2 = “sometimes”; 3 = “often”) how frequently their child exhibits a specified behavior (e.g., “has a short attention span”; “has trouble finishing tasks”). The 86 items fall into nine major constructs of executive functioning: *Inhibit*, *Shift*, *Self-Monitor*, *Emotional Control*, *Initiate*, *Working Memory*, *Plan/Organize*, *Task-Monitor*, and *Organization of Materials*. For each of the subscales, raw scores are transformed to age-and sex-adjusted T scores with a population mean of 50 and standard deviation of 10. Across all scales, higher T scores indicate more problematic behaviors within that domain.

The second neuropsychological questionnaire that parents completed was the Conners Attention Deficit/Hyperactivity Disorder (ADHD) Rating Scale, Third Edition (Conners, 2008). The Conners consists of 43 items that specifically probe symptoms of ADHD as defined by the DSM-5 (American Psychiatric Association, 2013). Parents rate on a 4-point scale (0 = “not true at all/never, seldom”; 1 = “just a little true/occasionally”; 2 = “pretty much true/often, quite a bit”; 3 = “very much true/very often, very frequently”) the degree to which each statement of true of their child, or how often it is true of their child (e.g., “has trouble concentrating”; “is hard to motivate”). The items can be combined into six constructs that are often used to describe symptoms of ADHD: *Inattention*, *Hyperactivity/Impulsivity*, *Learning problems*, *Executive Function*, *Aggression*, and *Peer Relations*. For each scale, raw scores are transformed into age-and sex-adjusted T scores with a population mean of 50 and standard deviation of 10. As was true for the BRIEF, higher T scores yielded from the Conners indicate more problematic behaviors in a given domain.

### 2.3 Home radon testing

Families were provided with a commercial short-term home radon testing kit.^2^ The test kit is a standard carbon-based envelope that hangs on an interior wall on the lowest livable level of the home for three to seven days. Of note, this type of test kit has shown good consistency with longer-term measurement tools (e.g., Ruano-Ravina et al., 2008; Li et al., 2023). After the testing period, the envelope is sealed and dropped in the mail for processing at the commercial lab. Parents were given the test kit along with instructions from the commercial vendor for proper exposure. We instructed families to leave the kit exposed for approximately 4 days. Our lab and the family each received a copy of the home radon results. In the case that a result exceeded the EPA action limit for mitigation (4 pCi/L), the principal investigator called the family to ensure they understood the results and provided additional information on radon safety and local resources. All kits were completed in Almanac-defined summer or winter periods as part of the original study design. We originally expected to detect significant fluctuations in home radon levels at different times of year and began the study providing families with two test kits – one to complete during the summer, and one to complete during the winter. However, after over 25% of the study sample completed kits during both seasons, we detected no significant seasonal fluctuations in measured home radon concentrations between summer (*M* = 7.24 pCi/L, *SD* = 7.09) and winter months (*M* = 6.83 pCi/L, *SD* = 5.82), *t* = 0.17, *p* = 0.87, *d* = 0.040. Given this information, the remaining participants only completed one short-term radon test kit. For participants who had two valid radon test results, we utilized their first measurement for analysis in the current investigation.

### 2.4 Computing a radon exposure index

Because the effects of radon exposure are cumulative, we computed a radon exposure index per participant, which yields results similar to international standards for computing cumulative radon dose as defined in the International Commission on Radiological Protection (ICRP) Publication 137 (Clement and Ogino, 2018). Additional details are provided in the Supplementary materials. The index was defined as the child’s home radon concentration (in pCi/L) obtained from the home testing kit multiplied by the amount of time they lived in that home (in years). That value was natural log transformed (see equation below), providing us with a normally-distributed index of chronic radon exposure in their current home.


$$RadonExposureIndex=\ln\left(\left[radonconcentration*exposuretime\right]+1\right)$$

For the present study, we divided the sample into three equal groups based on their radon exposure index values. The decision to explore radon exposure as a function of groups rather than as a continuous variable was based on prior literature indicating that the dose–response relationship between radon exposure follows a non-linear trajectory, with progressively stronger negative health outcomes as a function of increasing exposure (Duan et al., 2015). Thus, we had a low radon exposure group, a moderate radon exposure group, and a high radon exposure group.

### 2.5 Statistical analysis

We began by computing a principal components analysis (PCA) on the neuropsychological measures across the whole sample. Specifically, T scores obtained from the nine scales from the BRIEF and from the six scales from the Conners were used as input measures for the PCA. We assessed the Kaiser-Meyer-Olkin measure of sampling adequacy (KMO > 0.70) and Bartlett’s test of sphericity (significant at *p* < 0.05) to determine whether the data were well-suited to PCA. Then, components were selected based on varimax-rotated eigenvalues of at least 1.0. Component scores were saved per person for each of the resultant components, and these component scores were used for subsequent analysis.

Next, we computed a multivariate analysis of covariance (MANCOVA) wherein the component scores obtained from the PCA were the dependent variables. Radon exposure group (3-levels: low/moderate/high) was used as a between-subjects factor of interest to explore potential differences in neuropsychological profiles based on degrees of radon exposure. We also included age, sex, and SES as covariates in order to control for potentially confounding demographic factors. Of note, we did initially include a categorical indicating the season during which the radon test kit was completed (i.e., summer, winter), but this did not impact the results and was thus excluded from the final analyzes. We examined univariate effects for each component as well as *post hoc* analyzes with Bonferroni correction to further dissect specific effects by PCA component and by radon exposure group. Analyzes were conducted using SPSS version 25.

## 3 Results

### 3.1 Final sample and descriptive statistics

Of the 68 youths who were recruited to the study, 66 successfully completed at least one valid home radon test. We computed the radon exposure index for each participant by multiplying their obtained home radon concentration by the amount of time they had lived in the home, and natural log transforming the product. Youths were split into three equal groups (low, moderate, and high) based on their radon exposure index values. We then examined the T scores obtained from the BRIEF and Conners questionnaires for any potential outliers. Another six youths were excluded from analysis due to outlier data on one or more of the scales. Three of the excluded youths were originally part of the low exposure group, and another three were part of the moderate exposure group. Finally, one last participant was excluded for missing SES data.

The final sample in the study was comprised of 59 children and adolescents ranging in age from 6- to 14 years-old (*M* = 10.47 years, *SD* = 2.58; 28 males). Just over half of children in the study (53.3%) had home radon concentrations exceeding the EPA action limit of 4 pCi/L. Complete descriptives for all measures of interest for the full evaluable sample, separated by radon exposure group, are provided in Table 1.

**Table 1:** Descriptive statistics including sample demographics and T scores obtained from the BRIEF and Conners questionnaires for the overall sample and separated by radon exposure group.

	Full Sample (*n* = 59)		Radon exposure group					
			Low (*n* = 18)		Moderate (*n* = 19)		High (*n* = 22)	
	M	SD	M	SD	M	SD	M	SD
**Demographics**								
Age (years)	10.47	2.58	9.83	2.37	11.08	2.94	10.48	2.40
SES	48.88	7.46	52.37	7.98	48.15	7.18	46.67	6.48
Sex (M/F)	28/31		6/12		12/7		10/12	
**Radon exposure data**								
Radon Exposure Index	2.47	1.30	0.95	0.58	2.40	0.37	3.77	0.75
Radon Concentration (pCi/L)	6.62	7.35	3.24	3.62	4.62	4.21	11.10	9.44
Exposure duration (years)	3.87	3.23	1.21	1.07	4.60	3.70	5.70	2.47
**BRIEF T scores**								
Inhibit	50.25	8.99	51.00	9.10	46.84	6.87	52.59	9.94
Shift	50.49	10.50	47.50	6.96	48.58	8.40	54.59	13.27
Self-monitor	52.68	8.23	51.28	5.50	48.74	4.77	57.23	10.41
Emotional control	49.93	8.79	51.06	8.64	45.63	7.22	52.73	9.07
Initiate	50.47	8.69	51.11	7.05	46.85	7.26	53.00	10.24
Working memory	48.75	7.36	49.06	6.68	47.00	7.23	50.00	8.00
Plan/organize	49.19	7.79	50.50	6.10	46.32	8.78	50.59	7.77
Task-monitor	50.97	8.79	51.00	7.39	48.53	8.80	53.05	9.63
Organization of materials	49.81	6.89	48.44	4.13	49.49	8.25	51.50	7.35
**Conners T scores**								
Inattention	49.93	8.16	49.39	6.44	48.11	8.25	51.95	9.19
Hyperactivity/Impulsivity	56.44	11.22	55.83	11.52	55.32	9.89	57.91	12.37
Learning problems	48.44	7.16	47.06	5.45	49.79	6.87	48.41	7.18
Executive functioning	52.12	9.42	51.83	8.05	48.79	8.12	55.23	10.77
Aggression	50.39	8.48	48.00	5.49	47.63	3.83	54.73	11.42
Peer relations	51.88	10.26	49.56	6.35	49.00	7.35	56.27	13.10

### 3.2 Principal components analysis

We computed a PCA on the T scores obtained from the BRIEF and the Conners questionnaires. The data were well-suited to the PCA and met assumptions of the analysis scheme (KMO = 0.807; Bartlett’s test of sphericity: χ^2^(105) = 565.69, *p* < 0.001). The analysis yielded a three-component solution, which accounted for 66.87% of the total variance (see Table 2). Component 1 was comprised primarily of task-based executive functioning skills (*Working Memory, Plan/Organize, Task-Monitor, Organization of Materials, Inattention, Learning Problems,* and *Executive Functioning*). Component 2 was more oriented toward self-and emotional regulation abilities (*Shift, Self-Monitor, Emotional Control, Initiate, Aggression,* and *Peer Relations*). Component 3 was comprised of two scores pertaining to inhibitory control (*Inhibit* and *Hyperactivity/Impulsivity*). For each participant, we computed the component score for each of the three components and submitted them to the next analysis.

**Table 2:** Results of the principal components analysis with varimax rotation.

		Component 1	Component 2	Component 3
BRIEF Scales	Inhibit	0.231	0.453	**0.780**
	Shift	0.297	**0.727**	0.034
	Self-Monitor	0.168	**0.769**	0.259
	Emotional Control	0.345	**0.657**	0.338
	Initiate	0.545	**0.670**	−0.059
	Working Memory	**0.802**	0.309	0.201
	Plan/Organize	**0.687**	0.512	0.003
	Task-Monitor	**0.734**	0.326	0.009
	Organization of Materials	**0.770**	0.267	0.048
Conners Scales	Inattention	**0.658**	0.242	0.320
	Hyperactivity/Impulsivity	0.091	−0.012	**0.910**
	Learning Problems	**0.661**	−0.173	0.230
	Executive Functioning	**0.820**	0.245	0.005
	Aggression	0.014	**0.661**	0.238
	Peer Relations	0.201	**0.622**	−0.131
	Rotated Eigenvalue	4.42	3.71	1.90
	% Variance	29.49	24.75	12.63

Bolded values indicate the strongest PCA loading for a given variable.

### 3.3 MANCOVA on component scores

**Figure 1:**
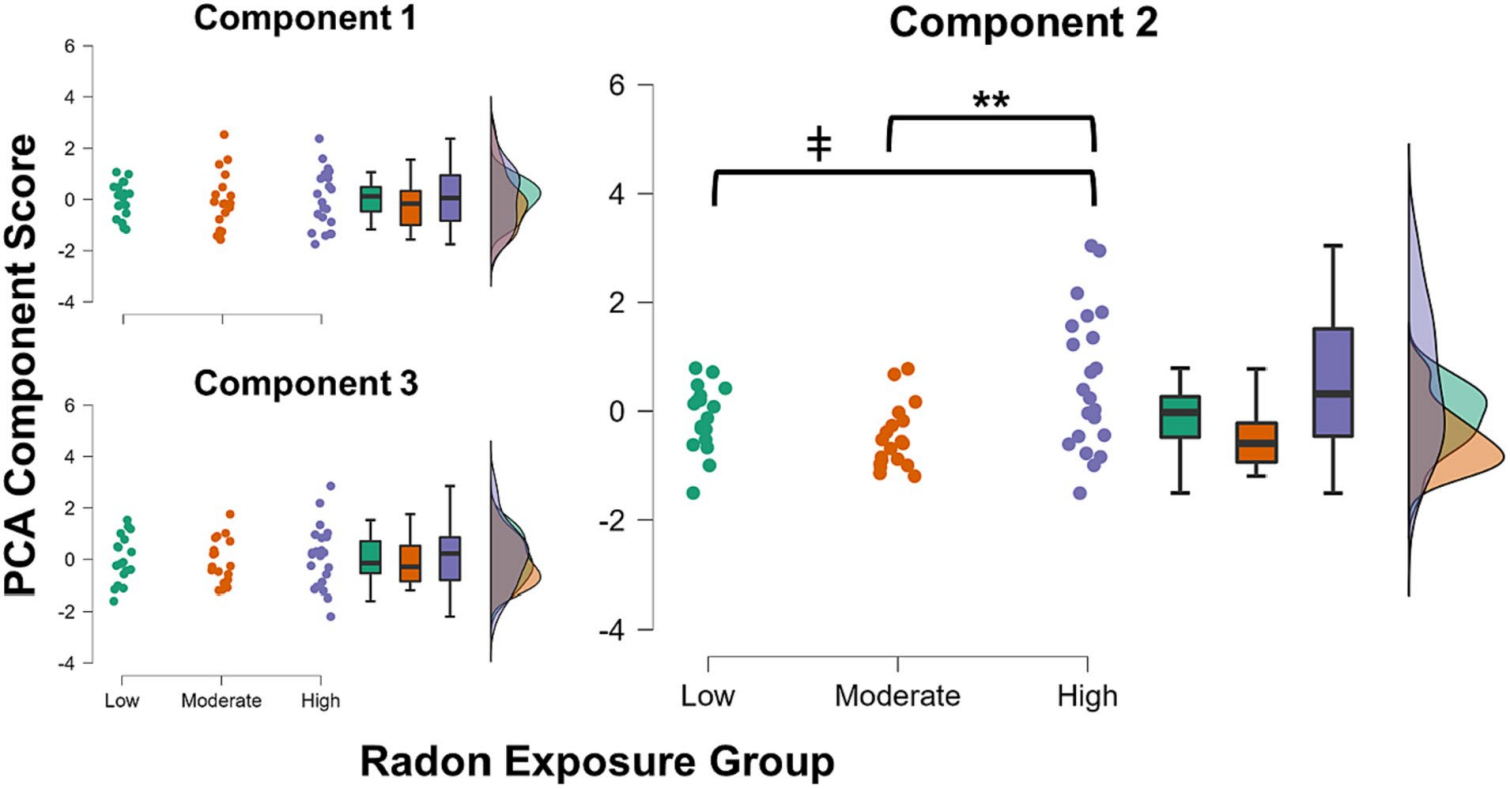
Raincloud plots showing distributions of each of the three obtained PCA component scores for each radon exposure group. There were no significant group differences in component scores for either Component 1 or 3. Youths in the high radon exposure group had significantly higher scores on Component 2 than youths in the moderate exposure group (pBonf = 0.002) and trended toward higher scores relative to youths in the low exposure group (pBonf = 0.07). ** *p* < 0.01, Bonferroni corrected; ǂ *p* < 0.10, Bonferroni corrected.

**Figure 2:**
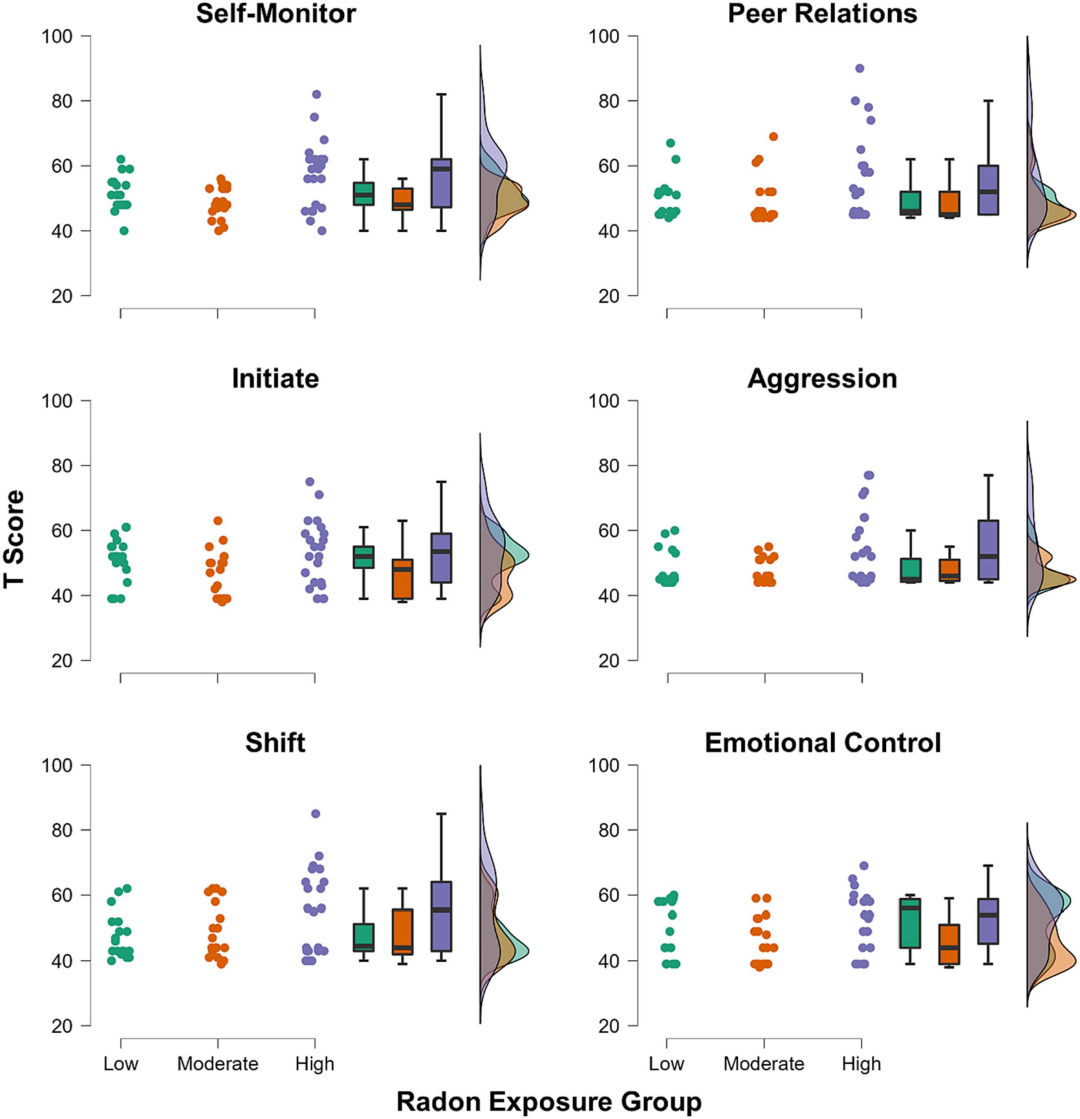
Raincloud plots showing groupwise distributions of T scores for each of the six subscales from the BRIEF and Conners that comprised the Component 2 score.

The three component scores yielded from the PCA were input as dependent variables in the MANCOVA. Radon exposure group (3-levels: low/moderate/high) was the between-subjects factor of interest, and we controlled for the effects of age, sex, and SES within the model. There was a significant multivariate effect of radon exposure group (λ = 0.77, *F*(6, 102) = 2.32, *p* = 0.038, η_p_^2^ = 0.12). Subsequent inspection of the univariate effects suggested that the effect was driven by group differences in Component 2 scores (*F*(2, 53) = 7.21, *p* = 0.002, η_p_^2^ = 0.21; Figure 1), with no significant group differences noted in either Component 1 or Component 3 scores (*p*s = 0.95 and 0.84, respectively). *Post hoc* tests showed that after Bonferroni correction, youths in the high radon exposure group had significantly higher scores on Component 2 relative to youths with moderate radon exposure (Δ*M* = 1.09, *p*_Bonf_ = 0.002), and trended toward having higher scores than youths in the low exposure group (Δ*M* = 0.74, *p*_Bonf_ = 0.07). For more complete visualization, Figure 2 shows the raincloud plots of T scores for the each of the six subscales from the BRIEF and Conners that comprised Component 2, separated by radon exposure group (see also Supplementary Figure S2). We did not detect any other statistically significant multivariate effects in the MANCOVA (*p*s = 0.10 to 0.68).

## 4 Discussion

In the present study, we investigated the effects of everyday home radon exposure on neuropsychological functioning in a sample of typically developing youths. Youths were divided into three groups based on their degree of chronic radon exposure, which was computed by integrating individual in-home radon concentration measures and the duration of time that the participant had been exposed to radon in that home. We then established domains of functioning across a set of well-validated parent-report measures of children’s cognitive, emotional, and behavioral well-being. The analysis derived a three-component solution, with domains generally representative of 1) task-based executive functioning, 2) self-and emotional regulation abilities, and 3) inhibitory control. We found that youths in the high radon exposure group showed significantly greater levels of parent-reported dysfunction in self-and emotion-regulation abilities when compared to youths in the middle-and lower-radon exposure groups. Importantly, these effects were above and beyond potentially confounding effects (e.g., age, socioeconomic status). We discuss the implications of our findings in greater detail below.

We originally hypothesized that we would find broad increases in neuropsychological dysfunction among youths as a function of greater radon exposure. However, we found more domain-specific effects, such that youths with the greatest degree of chronic home radon exposure showed significant decrements in only self-and emotion regulation abilities. This domain was comprised of measures of peer relations, emotional control, aggression, self-monitoring, initiation, and shifting. Self-and emotion-regulation faculties are generally considered to be transdiagnostic processes that are associated with a swath of mental health conditions spanning internalizing (e.g., depression, anxiety) and externalizing (e.g., defiance, physical aggression) symptoms and disorders (Cai et al., 2021). These data add to a rich literature exploring the effects of environmental toxin exposures on mental wellness.

Decades of research have demonstrated clear links between numerous exposures, including secondhand smoke, lead, and particulate matter and changes in cognitive abilities and mood regulation (Cohen et al., 2010; Dufford et al., 2021). For instance, in a longitudinal cohort study, Roberts et al. (2019) explored the effects of multiple air pollutants, including fine particulate matter (PM_2.5_) and nitrogen dioxide (NO_2_), on youths’ psychological well-being at two time points (age 12 and age 18). The study measured symptoms and diagnoses of depression, anxiety, ADHD, and conduct disorder at both time points, whereas air pollution was estimated only during the first time point. Although the authors did not detect significant links between concomitantly assessed air pollution and mental health outcomes at the first time point, pollutant exposures at age 12 did predict specific increases *only* in the risk of developing major depressive disorder at age 18 (Roberts et al., 2019). These findings are corroborated by a recent systematic review of adult studies, which demonstrated consistent increases in depression, anxiety, and suicidality among individuals who had more long-term exposure to two classes of fine particulate matter (PM_2.5_ and PM_10_; Braithwaite et al., 2019). This collection of studies not only emphasizes the specificity of inhaled air pollutants on certain aspects of mental health relevant to emotion regulation, but also demonstrates the importance of examining the *chronic* effects of toxic exposures on mental health. Further research is needed to better characterize the nature of chronic home radon exposure on more specific aspects of mental health in youths, including more specific symptomologies.

In addition, our data are well-aligned with the growing body of work showing potential links between indoor radon exposure and neurological health in aging populations. One such study quantified the concentrations of several specific radon progeny in postmortem brain tissue acquired from an older adult who had Alzheimer’s Disease (Momcilović et al., 2006). The authors noted significant concentrations of radon progeny distributed in several neural substrates, including the amygdala, hippocampus, and frontal lobes. Notably, the study found significant loss in cells, specifically in gray matter, as a function of greater concentrations of radon and progeny in these neural tissues (Momcilović et al., 2006). These findings are of great relevance to our current investigation given the large body of work implicating these specific neural substrates in self-and emotion regulation abilities (Cardinal et al., 2002; Barch et al., 2016; Picci et al., 2022). It is possible that everyday home radon exposure may be impacting sensitive structural development in critical neural substrates that underlie emotion regulation abilities, resulting in the noted dysfunction in observable behaviors in the present study. Importantly, prior work has demonstrated that minute individual-level variations over time in structures like the amygdala do reliably alter functional connectivity between brain networks that are strongly associated with mental health outcomes (Taylor et al., 2022). Thus, it is critically important to continue investigating the potential impacts of radon exposure on brain structure and function to further characterize how such toxic exposure affects developing youths.

Generally speaking, studies suggest that noxious exposures impact cognitive and mental health through multiple mechanisms including systemic inflammation which can cause damage to brain tissues (Trimble and Krishnamoorthy, 2000; Kleinman et al., 2008; Eisenberger et al., 2010; D’Angiulli, 2018, 2019). Recent work has demonstrated significant increases in specific inflammatory biomarkers in youths as a function of chronic home radon exposure (Taylor et al., 2023). Importantly, increases in these specific biomarkers, c-reactive protein (CRP) and interleukin (IL)-1β, have been linked with greater incidence and severity of psychiatric symptoms and disorders characterized by self-and emotion dysregulation (Farooq et al., 2017; Tabatabaeizadeh et al., 2018; Fourrier et al., 2019; Zainal and Newman, 2021). It is possible that radon-related increases in inflammation may, at least in part, explain the noted problems in emotion regulation in the current study. However, this is speculative and future work is needed to explore the potentially mediating effects of inflammation on the relationship between radon exposure and mental health outcomes.

Before closing, it is important to note several limitations of the current study. First, the current study used only a single short-term radon kit to quantify home radon exposure. Although these are commonly used testing devices, long-term kits would provide a more stable measurement of home radon concentrations. Additionally, our measurements were focused on each child’s most recent chronic home radon exposure. Incorporating more historical data about radon exposures from prior dwellings, as well as exposures in other frequently-visited buildings (e.g., schools) and more detailed indicators of time spent in each setting would certainly make for a richer picture of each individual’s chronic radon exposure. In addition to limitations in radon measurements, we relied on parent-reported neuropsychological measures. Including measures from other reporters, including teachers, clinicians, and the individual may yield varying results. Finally, the current study did not include measures of other potential concomitant environmental exposures. Home radon is one of many everyday exposures that youths are likely to encounter, and it may have unknown interactive effects with other toxins (e.g., NO_2_, PM_2.5_). Future investigations would benefit from exploring a richer profile of toxic exposures in parallel.

## 5 Conclusion

In conclusion, the current study is one of a select few that have examined the effects of chronic home radon exposure on youths’ mental well-being. Specific strengths of the study include the use of individual home measurements, rather than traditionally-employed methods of county-level estimates of radon concentrations, along with attention to the chronicity of the exposure. We found specific impacts of chronic home radon exposure on self-and emotion-regulation abilities, where youths with greater degrees of chronic home radon exposure exhibited greater dysfunction in emotional control, aggression, peer relations, and several other areas. Importantly, these effects were noted above and beyond the effects of potential confounds, including socioeconomic status. These data suggest that everyday home radon exposure may be impacting youths’ mental health and support the need for better public policy surrounding indoor radon mitigation efforts, and public awareness of the potential harm of home radon. Based on our findings, we recommend that the broader public make efforts to reduce indoor radon concentrations when possible, and/or limit the amount of time spent in dwellings for which radon concentrations cannot be readily reduced in order to promote neurocognitive health.

## Data availability statement

The datasets presented in this study can be found in online repositories. The names of the repository/repositories and accession number(s) can be found at: https://coins.trendscenter.org.

## Ethics statement

The studies involving humans were approved by Pearl Institutional Review Board. The studies were conducted in accordance with the local legislation and institutional requirements. Written informed consent for participation in this study was provided by the participants' legal guardians/next of kin.

## Author contributions

BT: Conceptualization, Data curation, Formal analysis, Funding acquisition, Investigation, Methodology, Resources, Supervision, Validation, Visualization, Writing – original draft, Writing – review & editing. HP: Formal analysis, Writing – review & editing. OS: Formal analysis, Writing – review & editing. DR: Data curation, Project administration, Writing – review & editing. HJ: Data curation, Project administration, Writing – review & editing. AC: Data curation, Project administration, Writing – review & editing. RG: Data curation, Writing – review & editing. TW: Funding acquisition, Investigation, Project administration, Resources, Supervision, Writing – review & editing.

## Footnotes

^1^ https://coins.trendscenter.org/

^2^ https://www.radon.com/
