# Supplementary material for: Effects of chronic home radon exposure on cognitive, behavioral, and mental health in developing children and adolescents
Source: Front Psychol. 2024 Feb 26;15:1330469. doi: 10.3389/fpsyg.2024.1330469 (PMC10925658; doi:10.3389/fpsyg.2024.1330469)
Supplement: Supplementary file 1 [file Data_Sheet_1.docx]

Supplementary Material

Effects of Chronic Home Radon Exposure on Cognitive, Behavioral, and Mental Health in Developing Children and Adolescents

Brittany K. Taylor, PhD^1,2,3,*^, Haley Pulliam^1^^,2^, OgheneTejiri V. Smith^1,2^, Danielle L. Rice^1,2^, Hallie J. Johnson^1,2^, Anna T. Coutant^1,2^, Ryan Glesinger^1,2^, and Tony W. Wilson^1,2,3^

^1^Institute for Human Neuroscience, Boys Town National Research Hospital, Boys Town, NE, USA

^2^Center for Pediatric Brain Health, Boys Town National Research Hospital, Boys Town, NE, USA

^3^Department of Pharmacology and Neuroscience, Creighton University, Omaha, NE, USA

*** Correspondence:**Brittany K. Taylor
[Brittany.Taylor@boystown.org](mailto:Brittany.Taylor@boystown.org)

# Computing the Radon Exposure Index versus Cumulative Dose

In this supplementary exploration, we compare our radon exposure index to another common means of assessing cumulative effects of radon exposure in order to establish the validity of our simplified scale. As described in the main text, in order to capture the potential cumulative effects of children’s most recent home radon exposure, we multiply the measured home radon concentration per peron by the amount of time they have lived in their home (in years). Given that this scale yielded some values less than 1.0, we add one to each score, then natural log transform the resultant values in order to achieve a normally-distributed scale of radon exposure (see Methods section 2.4).

Alternatively, the International Commission on Radiological Protection (ICRP) Publication 137 (Clement and Ogino, 2018) provides information for computing cumulative doses of radon on a per-person basis using a combination of numerous factors. These factors include the indoor radon concentration to which the individual was exposed, the duration of exposure (years of time spent in that location), the amount of time the individual spends in that location (e.g., hours per day), and specific constants that are critically linked to expected dosing given the physical aspects of radioactive nuclide decay, in this case, radon-222.

In our exploratory study, we were able to acquire data on individual home radon concentrations, as well as the duration of exposure to radon in that specific dwelling (in years). Although we did not acquire information on the number of hours each person spends in the home, we can reasonably estimate that at least 70% of a child’s time is spent in the home based on recent literature exploring the potential impacts of indoor pollutants on human health, though these numbers are likely higher (e.g., Klepeis et al., 2001; Stanley et al., 2019). However, we conservatively estimate 70% for the sake of this comparison between radon exposure metrics. In addition, based on guidance from existing publications (Marsh et al., 2010; Clement and Ogino, 2018), we also employed an occupancy dose factor of 9mSv/WLM, and an equilibrium factor as follows:

F = ([0.106*c_Po-218_]+ [0.514*c_Pb-214_]+ [0.318*c_Bi-214_]) / c_Rn-222_

Thus, we were able to compute a cumulative dose in millisieverts (msv) based on home radon concentrations per person for the study sample. Of note, the result was non-normally distributed and included values less than 1.0, thus we used the same strategy as noted above: we added 1.0 and natural log transformed each person’s cumulative dose for additional analyses.

To assess similarity of the two radon exposure scales (i.e., the radon exposure index and cumulative dose), we ran bivariate correlations between variable pairs of interest, as well as the variables that comprised those scales of interest.

# Results and Conclusions: the Radon Exposure Index versus Cumulative Dose

Descriptive statistics for the radon exposure index and associated variables are presented in the main text (see Results section 3.1). The average estimated cumulative radon dose for the sample was 34.119 ± 67.855 msv, and ranged from < 1.0 to 484.200 msv. After natural log transforming for normality, the scale ranged from 0.00 to 6.16 (*M* = 2.675, *SD* = 2.808).

Correlations between the radon exposure index and its counterpart, the natural log transformed cumulative dose, showed remarkably high correlations (*r* = 1.00, *p* < .001; see Table S1 and Supplementary Figure 1). Likewise, the untransformed values ([radon concentration * duration of exposure] correlated with untransformed [cumulative dose]) were also perfectly correlated, regardless of whether we used Pearson (*r* = 1.00, *p* < .001) or Spearman correlations (ρ = 1.00, *p* < .001) to compute the association.

**Table S1.** Correlations between the radon exposure index and cumulative radon dose, as well as variables comprising the scales.

|  | **1** | **2** | **3** | **4** | **5** | **6** |
| --- | --- | --- | --- | --- | --- | --- |
| 1. Home Radon Concentration (pCi/L) | – |  |  |  |  |  |
| 2. Duration of Exposure (years) | .012 | – |  |  |  |  |
| 3.Radon Concentration * Duration of Exposure | .686 | .458 | – |  |  |  |
| 4. Radon Exposure Index (ln) | .617 | .637 | .687 | – |  |  |
| 5. Cumulative Dose (msv) | .686 | .458 | 1.000 | .687 | – |  |
| 6. Transformed Cumulative Dose (ln) | .611 | .636 | .677 | 1.000 | .677 | – |


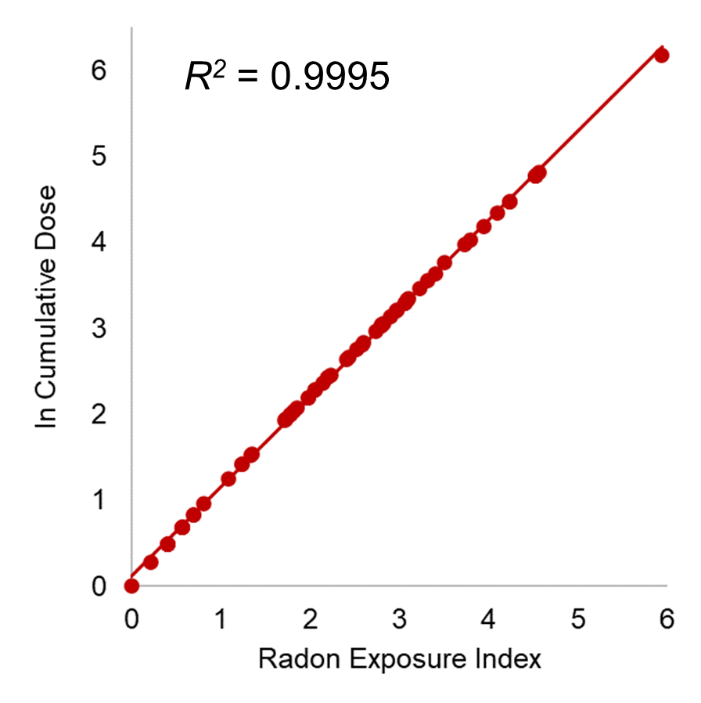


**Supplementary Figure 1.** Correlation between the radon exposure index as presented in the main text, and the natural log transformed cumulative radon dose.

Given the robust associations between the cumulative radon dose measures computed in accordance with international guidelines (Clement and Ogino, 2018), and our simplified radon exposure index presented in the main text, we conclude that the radon exposure index is a valid measure of cumulative radon exposure and can be reasonably used to assess long-term effects of home radon exposure.

# Individual Scales from Component 2

In Supplementary Figure 2, we show statistical comparisons by radon exposure group for each of the six subscales that comprised Component 2. Note that these statistical tests are uncorrected for multiple comparisons, and are displayed for transparency and future exploration purposes.


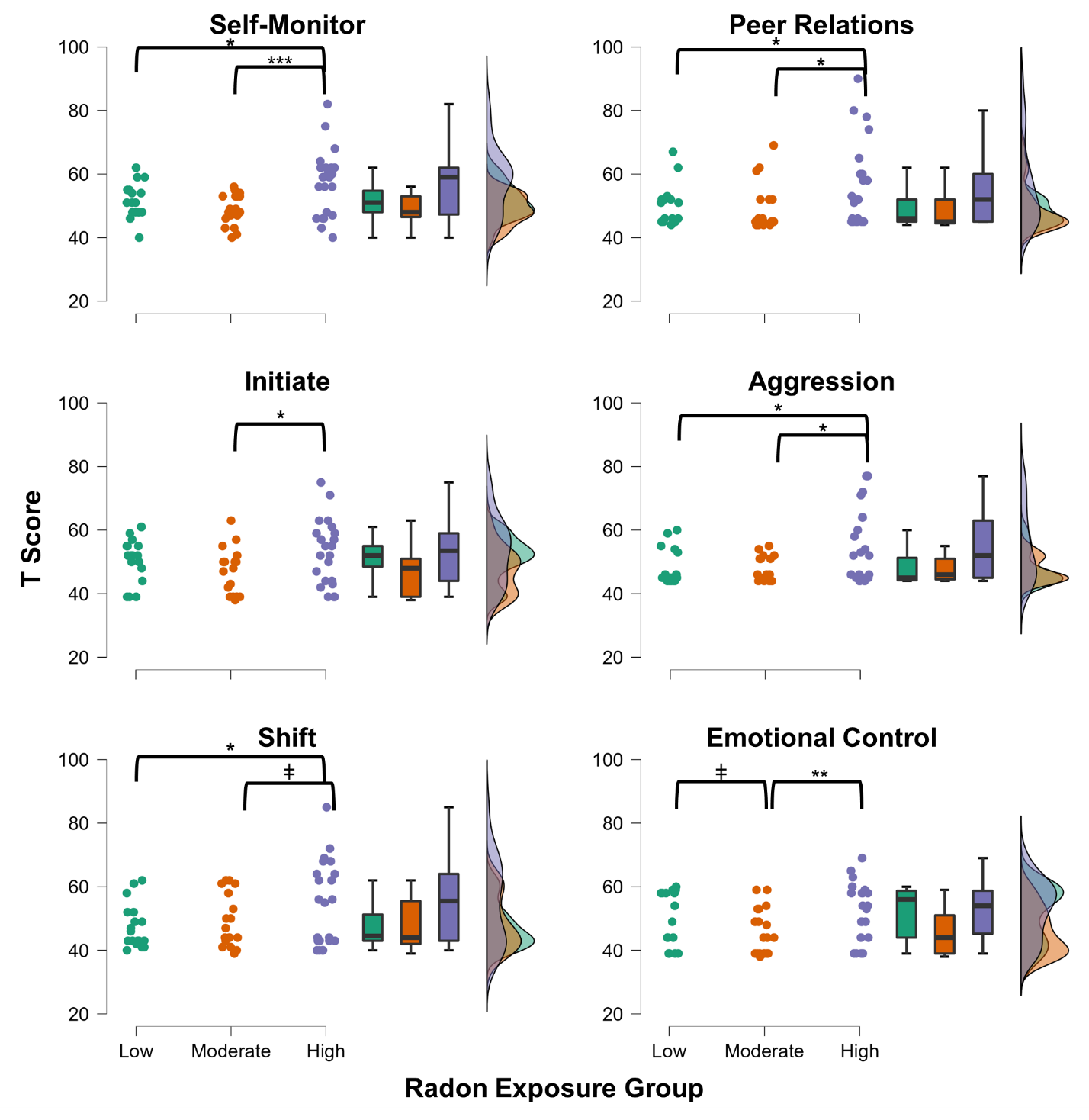


**Supplementary Figure 2.** Raincloud plots showing groupwise distributions of T scores for each of the six subscales from the BRIEF and Conners that comprised the Component 2 score. Note that all tests are uncorrected for multiple comparisons. * *p* < .05; ** *p* < .01; *** *p* < .001; ǂ *p* < .10

**References**

Clement, C., and Ogino, H. eds. (2018). “Occupational intakes of radionuclides: Part 3,” in *Annals of the ICRP* (London: SAGE), 491.

Klepeis, N. E., Nelson, W. C., Ott, W. R., Robinson, J. P., Tsang, A. M., Switzer, P., et al. (2001). The National Human Activity Pattern Survey (NHAPS): a resource for assessing exposure to environmental pollutants. *J Expo Sci Environ Epidemiol* 11, 231–252. doi: 10.1038/sj.jea.7500165

Marsh, J. W., Harrison, J. D., Laurier, D., Blanchardon, E., Paquet, F., and Tirmarche, M. (2010). Dose conversion factors for radon: recent developments. *Health Phys* 99, 511–516. doi: 10.1097/HP.0b013e3181d6bc19

Stanley, F. K. T., Irvine, J. L., Jacques, W. R., Salgia, S. R., Innes, D. G., Winquist, B. D., et al. (2019). Radon exposure is rising steadily within the modern North American residential environment, and is increasingly uniform across seasons. *Sci Rep* 9, 18472. doi: 10.1038/s41598-019-54891-8
